# Digitally-Mediated Social Stories Support Children on the Autism Spectrum Adapting to a Change in a ‘Real-World’ Context

**DOI:** 10.1007/s10803-020-04558-5

**Published:** 2020-06-09

**Authors:** Elizabeth Smith, Aurora Constantin, Hilary Johnson, Mark Brosnan

**Affiliations:** 1 Department of Psychology, University of Bath, Claverton Down, Bath, BA2 7AY UK; 2 School of Informatics, University of Edinburgh, Edinburgh, EH8 9AB UK; 3 Department of Computer Science, University of Bath, Claverton Down, Bath, BA2 7AY UK

**Keywords:** Autism, Social stories, Adapting to change, Real-world setting

## Abstract

Social Stories™ (SS) is a widely used intervention for children on the autism spectrum. A preliminary survey of 103 practitioners highlighted that SS are often used to support adapting to a change. This study investigated the use of digitally-mediated SS to support ten children on the autism spectrum attending a school summer camp. Teacher perceptions of anxiety, understanding and closeness to the goal of the SS were assessed before and after the intervention (prior to the event). The pre- post-intervention comparisons highlighted significant improvements in child understanding, anxiety, and closeness to goal with medium-large effect sizes. The child’s understanding and closeness to SS goal post-intervention related to their difficulties with the SS goal and their anxiety during the event.

## Introduction

Recent estimates suggest that approximately 1 in 59 children are diagnosed with autism, a lifelong neurodevelopmental condition characterised by impairments in social communication and interactions, combined with repetitive and restricting patterns of behaviour, activities and/or interests (RRBs) (American Psychiatric Association 2013; Baio et al. 2018). Males are diagnosed more than females with a ratio of around 4:1 (Baio et al. 2018). As children on the autism spectrum typically have impaired social skills they often lack the ability to understand social cues and protocols. Social skills are essential for both academic and social success, as they facilitate peer acceptance and student–teacher relationships (Garwood and van Loan 2019). Such impairments can be a cause of distress and frustration, and using parental/carer report, Brereton et al. (2006) identified greater disruptive, inattentive and anxious behaviour in children on the autism spectrum compared to children with intellectual disability. Research suggests that approximately two thirds of children on the autism spectrum display such behaviours (Hartley et al. 2008), which can have a negative impact upon daily activities (Brereton et al. 2006), create life-long barriers to inclusion (Rhodes 2014), and increase caregiver/family stress (Ludlow et al. 2011; Tomanik et al. 2004; Yacoub et al. 2018).

A popular intervention for children on the autism spectrum aimed at supporting social skills is Social Stories™ (SS). SS, first developed by Carol Gray in the 1990′s (Gray and Garand 1993), provide social information in a simple visual format that explains what to expect and what constitutes appropriate behaviour (Hutchins and Prelock 2013). They are highly structured, personalised social narratives that are developed and delivered in according to a set of specific criteria to ensure that the content has an emphasis on being descriptive rather than directive (Gray 2010, 2018). They have proved to be an acceptable intervention within the autism community and are widely used both within school and home settings (Green et al. 2006; Hess et al. 2008; Reynhout and Carter 2009).

However, despite their popularity, and an increase in the number of published studies within this area, the evidence relating to the effectiveness of SS remains questionable. Case studies have reported positive effects of using SS interventions (e.g. increasing play skills—Barry and Burlew 2004; appropriate behaviour at lunchtime—Bledsoe et al. 2003; or reducing disruptive behaviours—Crozier and Tincani 2005; Scattone et al. 2002). However, a large number of reviews and meta-analyses have now been conducted and these reveal a less positive picture overall (Ali and Frederickson 2006; Garwood and Van Loan 2019; Kokina and Kern 2010; Leaf et al. 2015; Mayton et al. 2013; McGill et al. 2015; Qi et al. 2018; Test et al. 2011). Such reviews yielded broadly similar conclusions, highlighting mixed findings in terms of effectiveness and raising a series of methodological weaknesses and reliance on single case study methodology. Kokina and Kern’s findings, for example, highlight the large variation in effectiveness across both studies and participants. The authors found that whilst 51% of included SS interventions were classified as “highly effective”, almost all the remaining stories (44%) were deemed “ineffective”. Similarly, McGill et al. (2015) found effect sizes to vary from small to large.

When determining the effectiveness of SS, a key factor to consider is what the SS is addressing. Kokina and Kern (2010) identify four categories that SS interventions are used for, namely: reducing negative behaviour, increasing positive behaviour, managing transitions/novel situations/anxiety, and teaching new skills (academic/functional). This is consistent with Reynhout and Carter (2009), who found that within an educational context, teachers report using SS to target a wide range of behaviours including decreasing challenging behaviours, teaching social skills, and assisting with changes and new routines. Within the research literature, reviews have shown that SS are predominantly used to decrease negative behaviours or increase positive behaviours (95%; Garwood and van Loan 2019; 86%; Kokina and Kern 2010) with some evidence suggesting that SS are most effective when targeted towards reducing negative behaviours (Hutchins and Prelock 2013; Kokina and Kern 2010; Qi et al. 2018).

There is less research on the third category of using SS to target managing transitions/novel situations/reducing anxiety, which makes up 9% of the SS literature^1^ (Kokina and Kern 2010; and no studies in this category were identified by Garwood and van Loan 2019). Qi et al.’s (2018) systematic review of SS identified studies that only fell under the categories of increasing positive behaviours/social communication skills and decreasing negative behaviours, and Wahman et al.’s (2019) systematic review only included the categories of increasing positive behaviour and reducing negative behaviour. Whilst the effectiveness of SS is largely evaluated upon research assessing increasing/decreasing behaviour, there is evidence that in practice SS are used for managing transitions/novel situations/anxiety reduction (Briody and McGarry 2005; Daly et al. 2010; Ivey et al. 2004; Marion et al. 2016; Morrison and Gullón-Rivera 2016). Reynhout and Carter (2009) reported that 87% of the teachers they surveyed used SS to support the introduction of changes/new routines (such as transition to school, or visits to the dentist). This is pertinent as difficulties in this area may relate to features such as resistance to change/insistence on sameness, which are RRB diagnostic features that are specific to autism (APA 2013; see Russell and Brosnan 2018). Previously reported studies within this area have been largely anecdotal. For example, Daly et al. (2010) describe the use of SS in preparing a child for an upcoming allergy test and Morrison et al. (2016) describe how SS could be used to help support siblings of patients in Neonatal Intensive Care Units. Similarly, a case study approach has been used to explore how SS can reduce levels of anxiety (O'Connor 2009), which is pertinent as co-occurring clinical levels of anxiety are also reported to be extremely high in autism (30–40%, Simonoff et al. 2008; Uljarevic et al. 2016). In addition, adapting to change has been proposed to be a separable aspect of RRBs that positively relates to anxiety in autism (Eisenberg et al. 2015; Uljarevic et al. 2016).

Thus within ‘real-world’ settings, there is evidence that SS are being widely used to support children on the autism spectrum to adapt to change (i.e. manage transitions/novel situations/reduce anxiety). However, little research on effectiveness focuses upon this. One challenge for research is how to assess the impact of the SS on supporting a child for an upcoming change. When SS are used to increase a positive behaviour or reduce a negative behaviour, the frequency of this behaviour can be compared pre- and post-intervention. This methodology, however, is less amenable to SS when the goal is to support the child manage a transition or adapt to a novel situation. In addition, increasing social skills or reducing challenging behaviours may be addressing behaviours that occur frequently and over an extended time period, whereas adapting to a specific change or event is likely to be relevant to a specific time point (such as visiting the dentist/allergy testing) and opportunities for comparison data can be limited. Teacher/parent ratings of perceived anxiety and closeness to the goal of the SS can be reliable measures within real world settings (Marshall et al. 2016).

In addition, it has been reported that researchers conform to Gray’s criteria for SS intervention more than teachers, when developing and delivering interventions (Mayton et al. 2013; McGill et al. 2015; Styles 2011) and little is known regarding intervention fidelity for SS within community settings (Mandell et al. 2013). Whilst evidence for efficacy (under ideal/controlled circumstances; see Singal et al. 2014) may be developed within a research-led context (Smith et al. 2020), any changes made within the real-world setting may compromise fidelity and effectiveness (Cohen et al. 2008; Gearing et al. 2011; Smith et al. 2007). This is pertinent as the high training requirements to ensure fidelity within real-world settings make this an unlikely solution to reduce disparities between research and practice (Stahmer et al. 2014). Significant variability in the implementation of evidence-based practices for children on the autism spectrum specifically have been identified, which highlights the need to address challenging issues related to fidelity in real-world settings (Mandell et al. 2013).

Children on the autism spectrum can show a preference for interventions being delivered through digital devices (such as iPads), compared to traditional methods, such as one sentence being written per page in a paper-based book (Bouck et al. 2014; Mancil et al. 2009; for systematic reviews of the benefits of iPads for autism interventions see Alzrayer et al. 2014; Kagohara et al. 2013). SS for children on the autism spectrum may be particularly amenable to delivery through digital technology (e.g. Kennedy et al. 2019; Ghanouni et al. 2019) as there are benefits of digitally-mediated SS for those who have difficulties with social interaction, enabling greater intensity of interaction with the content of the story. Digital technology can provide a more consistent and structured environment for the story, enabling repetition and direct feedback, and can offer the child more control over the learning experience. Digital technology can also enhance visual support, self-monitoring, and rewards, all of which can be personalised to the child (Constantin et al. 2017; Hanrahan et al. 2020; Moore 2008; Odom et al. 2003; Ozdemir 2010; Segers and Verhoeven 2005; Smith et al. 2020; Yildirim et al. 2001). Tablets, such as the iPad, have been used as a SS intervention for children on the autism spectrum through showing ‘social movies’ based upon the criteria for SS (Flores et al. 2014; Mowling et al. 2018). Almutlaq and Martella (2018) also used the ‘Kid in Story Book Maker’ app to successfully write a SS for three children on the autism spectrum (to give compliments).

Whilst digitally-mediated interventions have great promise for autism, evidence for best practice is yet to be established (Zervogianni et al. 2020a, b). Kim et al. (2018) reviewed nearly 700 mobile device apps listed under “Autism Apps” and found the 0.6% has direct evidence to support their usage. With respect to SS apps specifically, an online survey identified 22 features that parents and practitioners found desirable in a SS app (such as a library of stories, guidance of criteria for writing stories). A subsequent survey of app stores identified 19 SS apps and, of these, only one had more than half of these desired features (StoryMaker had 12/22; see Smith et al. 2020). SOFA-app was co-developed with the autism community (Constantin et al. 2017; see Parsons et al. 2019) and addresses all 22 desired features, and a prototype was used for the present study. The SOFA-app runs on mobile devices (smartphones and tablets) with Android and IOS operating systems (SOFA-app.org, released free-of-charge Autumn, 2020).

Thus the Stories Online for Autism (SOFA-app) application has been co-developed with the autistic community to enhance fidelity for community-delivered SS interventions through providing appropriate support for practitioners and parents/caregivers (Constantin et al. 2017; Hanrahan et al. 2020; Smith et al. 2020). An opportunity arose to use the SOFA-app with a group of children on the autism spectrum who needed support adapting to the same upcoming change. A school summer camp trip was planned for the class and the aim of the present study was therefore to explore the use of digitally-mediated SS for supporting children on the autism spectrum adapt to this upcoming change. Prior to the study, to put the research in a broader context, a preliminary survey of practitioners (who were familiar with using SS) was conducted to confirm the extent to which SS were used for adapting to change (i.e. managing transitions/novel situations/reducing anxiety) during their usual practice working with children on the autism spectrum in schools.

## Method

### Preliminary Survey

A brief online survey was conducted to identify the extent to which managing transitions/novel situations/ anxiety reduction was a goal for practitioners using SS in schools. The categories used in Kokina and Kern’s (2010); see also Reynout and Carter (2009) review (see below) were used in order to gauge a comparison between how SS are used in practice compared to the research literature. One hundred and three practitioners (96 female; 7 male) completed a brief online questionnaire. Participants were recruited via a range of autism-related online sources including: Autism Research (website for Autism Research); the National Autistic Society (UK Autism charity); Autism Speaks (US Autism charity); ASDTech (monthly newsletter for practitioners and parents interested in technology for ASD); EPNET (a forum used mainly by professionals within the area of Educational Psychology).

The majority (88.3%) of practitioners were based in the UK, with the remaining located in Malta, USA or Jersey. All respondents had experience of writing and/or delivering SS, and 61.7% of practitioners had received specific training for SS. The majority of practitioners (94.2%) reported using SS with children on the autism spectrum. Usage with children with Intellectual Disability (44.7%) and with those with co-occurring diagnoses (37.9%) were also common. A smaller group (17.5%) also used SS with typically developing children. The most popular age bracket was from 5 to 11 years (under 5 years = 25.2%, 5–7 years = 68.0%, 8–11 years = 68.0%, 12–16 years = 39.8%, 17 years +  = 12.6%).

The practitioners were asked if they had experience of using a SS within the following (non-exclusive) categories: (1) Reducing inappropriate behaviours; (2) Increasing appropriate behaviours; (3) Managing transitions/novel situations/reducing anxiety; (4) Teaching academic/functional skills. Results revealed that ‘Managing transitions/novel situations/reducing anxiety’ was the most common goal for SS (89.3%), followed by ‘Increasing appropriate behaviours’ (89.0%), ‘Reducing inappropriate behaviours’ (79.6%) and ‘Teaching academic/functional skills’ (29.1%). Whilst it is not possible to infer how representative this sample is of all practitioners, it does indicate that SS are widely used for supporting primary-aged (5–11 years) children on the autism spectrum adapting to change (consistent with Reynout and Carter 2009). The present study sought to identify the extent to which digitally-mediated SS could support a group of autistic primary school children adapting to an upcoming event.

#### Participants

Eleven children on the autism spectrum were invited to take part in the current study. All children attended a specialist autism unit within a primary school in the South West of England and had a formal diagnosis of Autism Spectrum Disorder from a clinician using established international criteria (APA 2013). Children without a functional understanding of English, or who were minimally verbal were not included. The Researcher (first author) worked with the Head of the autism unit to identify children who were going to attend the school summer camp and who met the inclusion criteria. One child was absent from school during the intervention week and therefore did not take part, leaving ten children (8 male; 2 female), aged 7–11 years (M = 9.3, SD = 1.49). Each child’s class teacher provided basic demographic details and evidence of the child’s attainment levels, according to UK National Curriculum teacher assessments—see Table 1 for full details.

**Table 1:** Child demographic and attainment details

Child characteristics	Frequency
Sex	
Male	8
Female	2
Clinical diagnosis	
Autism spectrum disorder	10
Intellectual disability/learning	0
Disability: other	0
Ethnicity	
White British	7
Asian	2
Dual heritage	1
National curriculum attainment levels	
Reading	
Y1 (age equivalent: 5–6 years)	3
Y2 (age equivalent: 6–7 years)	5
Y3 (age equivalent: 7–8 years)	2
Writing	
Y1 (age equivalent: 5–6 years)	6
Y2 (age equivalent: 6–7 years)	4
Y3 (age equivalent: 7–8 years)	0
Numeracy	
Y1 (age equivalent: 5–6 years)	2
Y2 (age equivalent: 6–7 years)	4
Y3 (age equivalent: 7–8 years)	4

UK teacher assessments provide levels that indicate the child’s attainment in reading, writing and numeracy relating to age equivalent norms

In addition, the same class teachers rated the presence or absence of autistic behaviours on the Social Communication Questionnaire (SCQ: Rutter et al. 2003). The SCQ Lifetime is a 40-item questionnaire which has two aspects. Firstly, respondents are asked to indicate whether a range of autistic behaviours have ever been present (or not) and secondly to indicate whether behaviours were present at age 4 years (or not). The SCQ Lifetime has a cut-off of 15 (from the 40 items), and typically developing school children have a mean of 3.89 (SD = 2.77) and a mode of 1 (see Mulligan et al. 2009; Chesnut et al. 2017). As the children were not at the school at age 4, only the first aspect of the SCQ Lifetime was rated by teachers (items 2–19), to confirm the presence of autistic behaviours in the present sample from the age of 4 onwards whilst they had been at the school (i.e. during the past 3–7 years). The mean for SCQ Lifetime (items 2–19) was 8.70 (SD = 3.12; range = 4–14). This confirmed that more autistic behaviours were present in the children on the autism spectrum than would be expected in typically developing children (one sample t-test (t(9) = 8.78, p < .0001: Note, this mean is below the cut-off of 15 for the full 40-item SCQ, as only the first aspect of the SCQ was used, items 2–19).

Parental consent and child assent were obtained. Full ethical approval from the University of [anonymised] Research Ethics Committee was obtained for this study.

#### Design

One of the challenges researching SS for adapting to change is that pre- and post-measures of behavioural frequency are not amenable to this situation. A range of measures were therefore developed over three timepoints: (1) Pre-intervention: Before a 1-week SS intervention; (2) Post-intervention: At the end of a 1-week SS intervention (and before the summer camp); and (3) Post-event: At the end of the summer camp. The 1-week intervention occurred in school, Monday–Friday the week before the summer camp. The summer camp then ran for 4 days (Tuesday–Friday) the following week. The three timepoints are therefore: pre-intervention, post-intervention, and post-event. Where appropriate, the study identified the effectiveness of the intervention through two analyses: (1) pre-intervention to post-intervention, and (2) post-intervention to post-event, using the measures below. The child’s class teacher completed all the questionnaires for the child in their class.

#### Measures

##### Summer Camp Questionnaire

Six elements, relating to the upcoming school summer camp, were identified by the Head of the autism specialist unit as potential causes of anxiety: (1) Trip to Puxton Park; (2) Swimming; (3) Trip to Cheddar Gorge; (4) Mealtimes; (5) Sleeping away from home/being away from family; (6) Storytelling group activity. These were used to make the Summer Camp Questionnaire, which comprised six 7-point likert scales (1 = not at all apprehensive; 7 = extremely apprehensive) to assess levels of perceived child anxiety related to each of the above elements/activities.

##### SS Questionnaire (Pre- and Post-intervention Versions)

Comprised two questions, each rated on an 11-point scale. The first question relates to the child’s closeness to the SS goal (on a scale of 0–10, please circle the number that best describes how close the child is to reaching his/her goal today [0 = goal not met, 10 = goal reached]; after Marshall et al. 2016). The child’s individual goal was written at the top of the questionnaire as reference (e.g. to feel well prepared for the trip to Barton Camp, specifically relating to mealtimes). The second question related to the child’s understanding of the SS goal (e.g. on a scale of 0–10 please circle the number that best describes the child’s level of understanding relating to what will happen during mealtimes at Barton Camp [0 = very poor understanding, 10 = excellent understanding]). The post-intervention version included an additional question in order to provide a comparison for the baseline anxiety measure within the Summer Camp Questionnaire. This question used the same 7-point anxiety scale as was previously used within the Summer Camp Questionnaire (e.g. how concerned/anxious was the child regarding the above situation [1 = not at all apprehensive, 7 = extremely apprehensive].

##### Post-event Questionnaire

This comprised two questions relating to the class teacher’s perception of the child’s experience during the summer camp itself. The first question asked about perceived problem severity and the second about perceived anxiety levels. Both questions were rated on an 11-point scale and were targeted at the activity that was the focus of their individual SS (e.g. ‘During his time at summer camp, how much of a problem did [name] have during the swimming activities?’ [0 = no problem at all, 10 = major problem]).

##### Child Smileyometer Questionnaire

Ratings on six smileyometers (Read 2008) corresponding to the six activities/elements within the adult Summer Camp Questionnaire. Children were asked to circle the appropriate face based on a 5 point scale (1 = awful, 2 = not very good, 3 = good, 4 = really good, 5 = brilliant) for each activity. An adult provided support with reading where required.

##### SOFA-app Questionnaire

A short pictorial questionnaire to assess the children’s experience of using the SOFA-app. For full detail see Table 2.

**Table 2:** Results from the child measures

Question	Frequency	Percentage
Story Smileyometer scores (5 days)*		
Brilliant	24/48	50.0
Really good	11/48	22.9
Good	13/48	27.1
Not very good	0/48	0
Awful	0/48	0
How easy did you find using the app?		
Easy	8	80.0
Not sure	2	20.0
Difficult	0	0
Would you prefer to read your story on the iPad or as a book?		
iPad	10	100
Book	0	0
Would you choose to use the SS app again?		
Yes	5	50.0
Maybe	5	50.0
No	0	0
Would you tell a friend to use the SS app?		
Yes	4	40.0
Maybe	6	60.0
No	0	0
How helpful did you find the SS app? (smileyometer)		
Brilliant	5	50.0
Really helpful	2	20.0
Good	3	30.0
Not very helpful	0	0
Awful	0	0

*Scores from the children’s ratings of their SS taken after each time the SS was read (approx. 5 times per child n = 48 due to 1 child missing the story once and 1 missing smileyometer rating)

#### Procedure

Prior to the intervention the Researcher met with the Head of the autism specialist unit to identify an appropriate upcoming event that may be anxiety provoking for the children, and for which it was felt that a SS could be of benefit. The school summer camp met this criterion and the ‘Summer Camp Questionnaire’ was developed. Copies were given to the child’s teacher to complete in order to identify an individual target goal for each child’s SS (the element/activity with the highest score). Prior to the intervention the child’s teacher also completed the brief SS Questionnaire and the child completed the Smileyometer Questionnaire. An individual SS was created using a prototype version of the SOFA-app on an iPad. Each child read their SS on the iPad with the Researcher once a day during the week before the school summer camp (total = 5 times). After each time the SS was read the child selected a rating on the smileometer (awful, not very good, good, really good, brilliant) as to their experience of reading the story. At the end of the week the class teachers also repeated the SS questionnaire (post-intervention version). After the summer camp, the class teachers completed the ‘Post-Event Questionnaire’. The children also repeated the smileyometer questionnaire and completed a short questionnaire on the SOFA-app.

#### Intervention (Fidelity)

The Researcher (first author; an Educational Psychologist with specific SS training) developed a SS for each child. The goal of the story was identified in collaboration with the child’s class teacher and related to the area that the child was perceived to be most apprehensive of, as rated in the Summer Camp Questionnaire. Carol Gray’s guidelines (Gray 2018) were followed both during the story development and implementation using the SOFA-app. After the child had read their story for the first time their comprehension was checked by asking three questions requiring the child to identify the correct response or missing word (e.g. How many nights will you be staying at Camp? [1, 2, 3, 4, 5]). Stories were reviewed and adapted throughout the intervention phase according to any feedback from the child. For example, on one occasion during reading the story the child commented ‘I don’t want to feel homesick’ in response to the text that read ‘It is okay if I miss my family and my dogs. Other children will probably miss their families too. This is called being homesick’. This was subsequently amended by removing the last sentence 'this is called being homesick', after which the child was happy with the story.

The number of SS per category were: Swimming (n = 1); Mealtimes (n = 2); Storytelling group activity (n = 3); and Sleeping away from home/ being away from family (n = 4). The two trips (to Puxton Park and Cheddar Gorge) identified as potential causes of anxiety by the Head of the specialist autism unit were not identified as sources of apprehension for the children. The SS were personalised for each child, even within the same category. For example, the SS for 'sleeping away from home' explained who else would be sleeping in the child's room with them and included their chosen item that were going to bring with them from home.

#### Data Analysis

As numbers were small, an exploratory analysis was conducted using non-parametric tests were used, including Wilcoxon Signed-Rank Test (W) to identify differences in pre- and post-intervention scores relating to the teachers’ perception regarding the child’s: (a) closeness to the SS goal; (b) perceived level of understanding relating to the SS goal; and (c) perceived levels of anxiety. Partial eta squared (η^2^) were calculated to indicate effect sizes (as a rule of thumb, 0.3+ is considered a medium effect size and 0.5+ a large effect size; Cohen 1988). The post-intervention scores were then correlated with the two outcome measures within the Post Event Questionnaire to consider their relationship with the child’s actual experience during the camp using Spearman’s rank correlation coefficients (r_s_). Finally, descriptive findings are presented from the children’s SOFA-app Questionnaire and their daily smileyometer ratings.

## Results

**Fig. 1:**
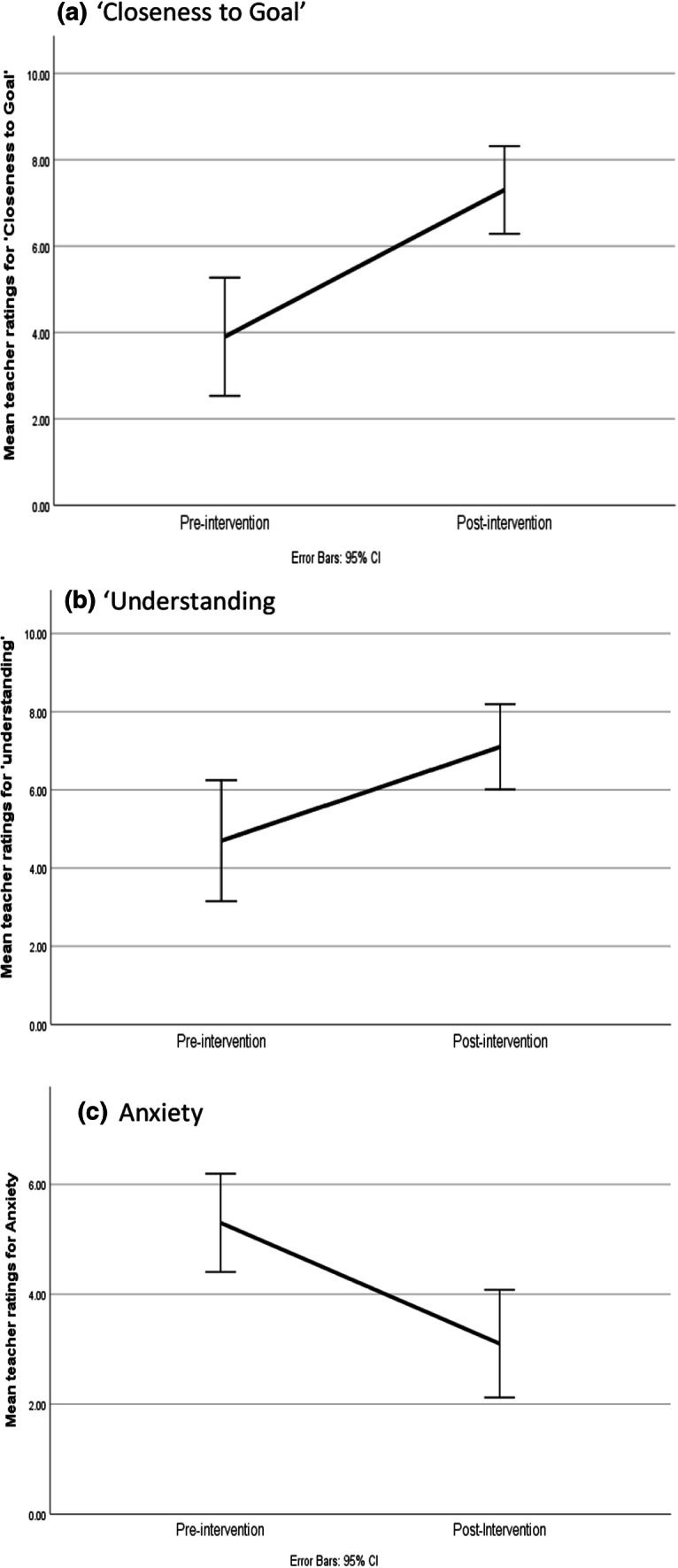
Graphs displaying mean scores for teacher ratings relating to their perception of the child’s percived ‘closeness to goal’, ‘understanding’ and ‘anxiety’ levels at pre- and post-intervention time points

Initially pre- and post- intervention scores were compared, revealing that that the children were significantly closer to reaching their goal at the post-intervention time point (M = 7.30, SD = 1.42), compared to pre-intervention (M = 3.90, SD = 1.91, W(10) = 2.68, p = 0.007; η^2^ = 0.60, see Fig. 1a) with 9 (out of 10) children responding positively. The children’s perceived level of understanding was also rated significantly higher post-intervention (M = 7.10, SD = 1.52), compared to pre-intervention (M = 4.70, SD = 2.16, W(10) = 2.09, p = 0.036; η^2^ = 0.47, see Fig. 1b) with 8 (out of 10) children responding positively. Perceived anxiety scores were also significantly lower post intervention (M = 3.10, SD = 1.37), compared to pre-intervention (M = 5.30; SD = 1.25, W(10) = 2.68, p = 0.007; η^2^ = 0.60, see Fig. 1c) with 9 (out of 10) children responding positively.

Secondly the post-intervention ratings were compared with the post-event ratings to identify any relationships between teacher ratings at the end of the intervention (but before the event) with teacher ratings of the behaviour during the event itself. During the event (i.e. the summer camp) the mean perceived difficulty the child was having with their SS goal (problem severity) was 4.0 (SD = 2.67, range = 1–8) and the mean perceived anxiety was 2.8 (SD = 1.48, range = 1–6). Correlations between each of the three post-intervention scores (closeness to goal, perceived understanding, perceived anxiety) and the two post-event scores (problem severity, anxiety) were conducted. Results revealed a significant negative relationship between the ratings for both ‘closeness to goal’ and ‘perceived understanding’ at post-intervention with problem severity (r_s_ = − .82, p = .004; r_s_ = − .67, p = .03; respectively) and perceived anxiety (r_s_ = − .77, p = .01; r_s_ = − .74, p = .02; respectively) during the summer camp. This indicated that the closer the child was perceived to be towards understanding and reaching and their goal, the less severe problems and anxiety they experienced during the summer camp. Perceived anxiety at post-intervention did not significantly correlate with problem severity or anxiety during the event (both p > .05). Importantly too, problem severity or anxiety during the event did not correlate with any of the pre-intervention measures (closeness to goal, perceived understanding, perceived anxiety, all p > .05).

Children were also asked to reflect on how they felt about the focus topic of their SS, identified by their Teacher from the Summer Camp Questionnaire (e.g. mealtimes). Based on their smileyometer scores (1 = awful, 2 = not very good, 3 = good, 4 = really good, 5 = Brilliant), the pre-intervention mean rating was 3.45 (SD = 1.01, range = 2–5) and the post-event mean was 4.15 (SD = 1.13, range = 2–5). Although this was a positive increase with a medium effect size, it was not significantly different (W(10) = 1.45, p = .15; η^2^ = 0.32). Finally, children completed an evaluation of the SOFA-app (see Table 2, which also has the children’s daily ratings as the top item).

## Discussion

SS is a widely used intervention for children on the autism spectrum, although the evidence for their effectiveness is mixed. In line with previous research (Reynhout and Carter 2009; Varnava et al. 2019), our preliminary survey confirmed that SS are often used to support adapting to change, that is managing transitions, novel situations, reducing anxiety, within primary education (ages 5–11). This is pertinent as the evidence for the effectiveness of SS largely draws upon research focussed upon reducing negative behaviour or increasing positive behaviour (Garwood and van Loan 2019; Kokina and Kern 2010; Qi et al. 2018). Difficulties adapting to change are part of the RRB diagnostic criteria for autism (APA 2013) and high levels of anxiety co-occur in many people on the autism spectrum (O’Nions et al. 2018; Simonoff et al. 2008; Uljarević et al. 2017). Adapting to change has been proposed to be a separable aspect of RRBs that positively relates to anxiety in autism (Eisenberg et al. 2015; Uljarević et al. 2017). This was the first study to examine the effectiveness of digitally-mediated SS in supporting a group of children on the autism spectrum adapting to change, as previous research has comprised of single case studies (Briody and McGarry 2005; Daly et al. 2010; Morrison and Gullón-Rivera 2016).

Findings indicated that a daily SS intervention, delivered over a 1-week period, significantly reduced perceived anxiety levels and increased understanding in the children prior to the change (attending a school summer camp). The children were also rated as being significantly closer to their target goal by the end of the intervention. The comparison of the pre- and post-teacher measures were therefore all positive, which was consistent with the child-based ratings. The effect sizes for the intervention were found to be medium to large. In addition, the relationships between the post-intervention ratings (before the event itself) and the child’s subsequent perceived experience during the summer camp indicated that these positive effects extended beyond the intervention phase, to the event itself. The greater a child’s understanding and reaching the social story goal prior to the event, the less problem severity and anxiety were displayed by the child during the event. Thus, despite the apparent dearth of SS research within the category of ‘managing transitions/novel situations/reducing anxiety’ (Kokina and Kern 2010; see also Garwood and van Loan 2019; Qi et al. 2018), this study indicates that SS are beneficial within this context. This is particularly pertinent as the preliminary survey (above) identified this as the most frequent category for SS interventions by practitioners.

Supporting the child’s understanding of the situation is central to a SS which should ‘provide a student with autism an accurate understanding of the situation in which the targeted behaviour occurs’ as they may be ‘more impaired in their access to accurate social information than their ability to understand and respond appropriately to it’ (Gray and Garand 1993: 2). A theoretical understanding of why SS can be effective needs to be developed and one potential is the Dual Process Theory of Autism which proposes that difficulties with rapid, implicit, spontaneous processing can be ameliorated by building upon strengths associated with slower, explicit, prompted processing (Brosnan et al. 2016, 2017; Lewton et al. 2019; see also Callenmark et al. 2014; Senju 2013; see Happé et al. 2017, for review).

Previously, a short protocol of less than ten sessions had been highlighted as impacting upon effectiveness (Kokina and Kern 2010). Our findings suggest that an intervention period of 1-week, consisting five sessions only, was sufficient to elicit a positive change. This is also of notable benefit considering time and budget constraints often reported by teachers (Lang et al. 2010; Machalicek et al. 2007). Within the SS literature previous studies have yielded small to large effect sizes (see McGill et al. 2015). The large effect sizes found within the current study may be due in part to the high level of fidelity as the Researcher developed and delivered the intervention (see McGill et al. 2015). In addition, they may also relate to the measures employed, which were kept deliberately short and simple for teachers to complete, in line with feedback from the largest SS evaluation feasibility study, and included the ‘goal based measure’ that was found to be most reliable (Marshall et al. 2016).

The findings suggest that digital SS can be effective in supporting children on the autism spectrum adapting to change through identifying the specific goal of the intervention and increasing understanding of the issue. This is of particular interest to autism, as the point of any intervention is to affect change, yet a diagnostic feature of autism RRBs is resistance to change/ insistence on sameness.^2^ Whatever the focus of the intervention, resistance to change may represent an obstacle to engagement for autistic participants (e.g. Isenberg et al. 2019). This would suggest that supporting the adaptation to change could be an invaluable addition to any intervention protocol. SS may therefore be a useful addition in preparing children on the autism spectrum for an upcoming intervention, as well as changes such as holidays or events such as visits to medical professionals.

It is also pertinent that children perceived the SS intervention to be a positive experience. All the child ratings were good to brilliant, and there were no negative responses to the other items, though it should be noted that the smileyometer has three positive and two negative options. Consistent with previous research (Bouck et al. 2014; Mancil et al. 2009), all the children indicated that they would prefer digitally-mediated SS intervention compared to traditional book formats, which may also have impacted upon the success of the intervention. A limitation is that the study did not index the children’s experience with traditional SS formats and this may reflect a general preference for using digital technology rather than a specific preference for digitally-mediated SS interventions. Digital technology has been argued to support a consistent, structured, personalised learning environment enabling repetition, direct feedback and control over the learning experience (Constantin et al. 2017; Hanrahan et al. 2020; Moore 2008; Odom et al. 2003; Ozdemir, 2010; Segers and Verhoeven 2005; Smith et al. 2020; Yildirim et al. 2001). The extent to which these features support a preference for slower, explicit, prompted processing in autism (see Brosnan et al. 2016, 2017), provides a framework for future research to identify whether digitally-mediated SS interventions are particularly effective for children on the autism spectrum. The SOFA-app has been co-developed with the autistic community with the aim of supporting the fidelity of the intervention. Whilst the present study followed the progress of children in a real-world context of a school summer camp, fidelity of the intervention was ensured by a Researcher developing and delivering the intervention. Other research is needed to identify the extent to which fidelity can be supported by the SOFA-app when the SS are developed and delivered by parents and teachers (see Smith et al. 2020).

When considering the findings from this study however, the following should be borne in mind. A major limitation is the small sample size and the number of comparisons, indicating the findings should be considered exploratory. The present study, however, is one of the largest studies of SS to support adapting to change in children on the autism spectrum, expanding upon previous research which has largely been based on case studies (see Qi et al. 2018). All the children had formal diagnoses from clinicians employing established criteria for Autism Spectrum Disorder (APA 2013). ADOS scores, were not available to the Researchers yet diagnosis was consistent with the SCQ assessment, though it should be noted that teachers were not able to reflect on the child’s behaviour before the age of 4.

As noted above, there are methodological issues which make this a challenging function of SS interventions to research. Firstly, there are no objective measures of behaviour change, the present study relied upon teacher ratings and it is possible that these may be susceptible to bias. It is not possible within this context to obtain objective pre- and post-measures of behavioural frequency, for example. Importantly too, there was no comparison data, we do not know what the problem severity or anxiety at the event would have been like without the intervention. In addition, without comparison data, firm conclusions cannot be drawn as to what impacted upon effectiveness. Whilst the findings are consistent with children on the autism spectrum having a preference for using digital technology and SS being effective, this study is limited in causal inferences that can be made. It may be the personal attention from the Researcher related to effectiveness for example. In their review of evidence for SS, Wright et al. (2016) identified seven between-groups SS published studies, all of which have methodological limitations. Importantly too the teachers and children were not blinded and were aware of the nature of the study. Recently, Hanrahan et al. (2020) identified benefits of SS when compared to a control condition of reading the child a poem.

Whilst post-intervention measures correlated with the summer camp assessments, the pre-intervention measures did not. This is potentially consistent with the intervention impacting upon the summer camp, although clearly post-intervention ratings were closer in time to the summer camp than the pre-intervention ratings. Larger numbers of participants would enable randomised control trials of the intervention, although larger numbers of children on the autism spectrum adapting to the same change at the same time may be difficult to identify. The present study addressed this through identifying a range of potential goals for the SS that may arise during the summer camp. Transitioning to school may represent a context that this approach would be useful for in future research.

Finally, the SS literature has focussed on increasing positive behaviour and reducing negative behaviour (e.g. Wahman et al. 2019) and this study has highlighted the effective use of SS for supporting adapting to change. The use of SS for teaching new skills (academic/functional) is an under-researched area (Garwood and van Loan 2019; Kokina and Kern 2010). As 29.1% of survey respondents in the present study indicated that they used SS for this purpose, future research can address the effectiveness of SS for teaching new skills.

## Footnotes

1 The use of SS for teaching new skills (academic/ functional) makes up the remaining 5% of research studies (Garwood & van Loan, 2019; Kokina & Kern, 2010).

2 https://www.cdc.gov/ncbddd/autism/hcp-dsm.html
